# Supplementary material for: In Vitro Organoid-Based Assays Reveal SMAD4 Tumor-Suppressive Mechanisms for Serrated Colorectal Cancer Invasion
Source: Cancers (Basel). 2023 Dec 13;15(24):5820. doi: 10.3390/cancers15245820 (PMC10742020; doi:10.3390/cancers15245820)
Supplement: Supplementary file 1 [file cancers-15-05820-s001.zip › Supplemental Figures.pdf]

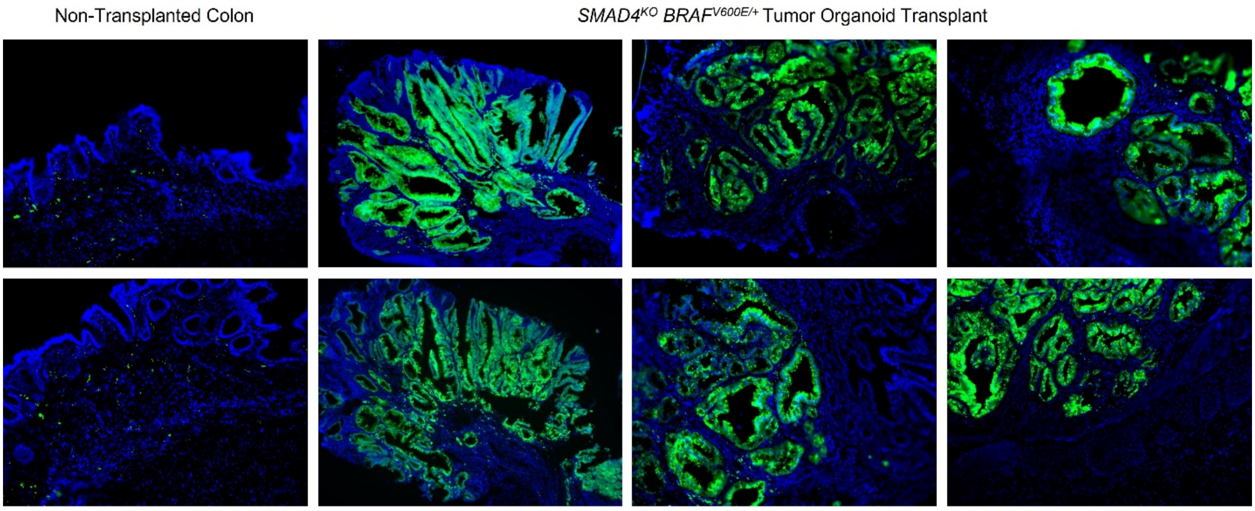

Supplemental Figure S1. Tumor organoids show invasive behavior *in vivo*. Representative images of orthotpically transplanted *Smad4*^{KO} *BRAF*$^{V600E/+}$ organoids. Scale bars = 0.05mm

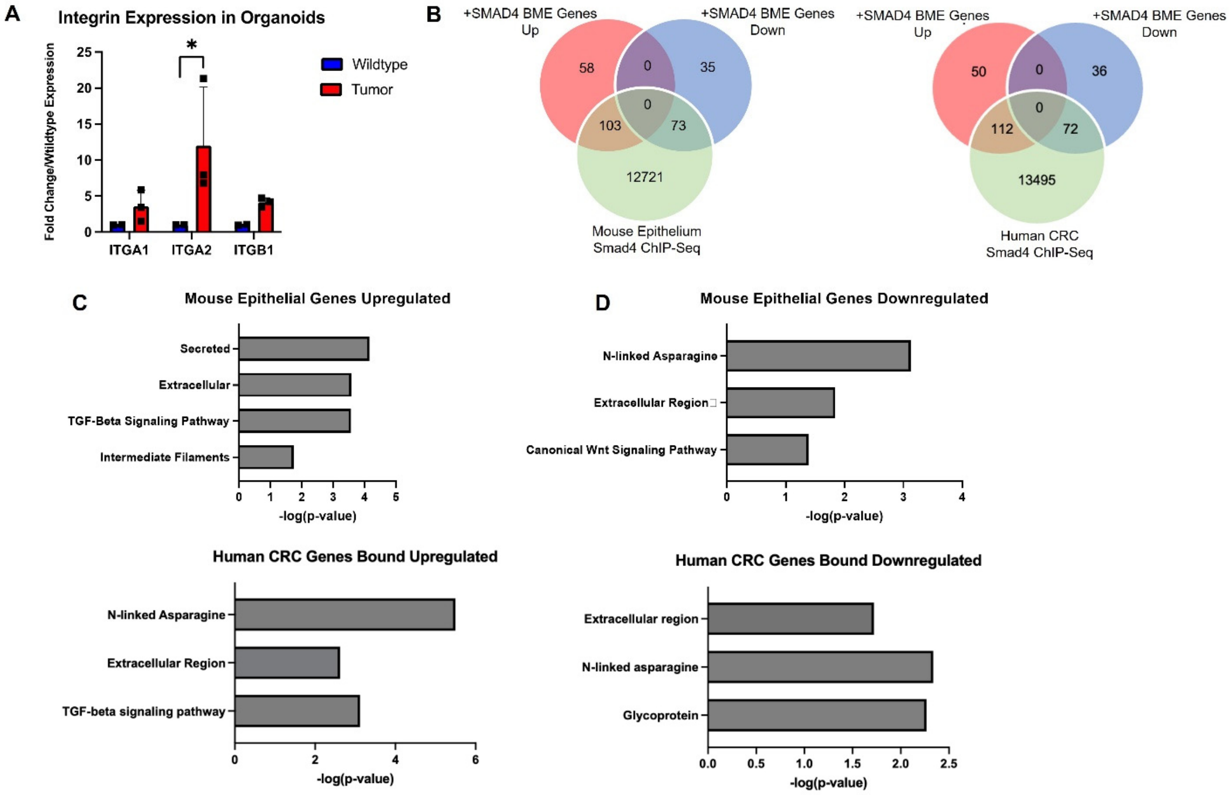

Supplemental Figure S2. SMAD4 is enriched at ECM and Secretory Pathway Genes. A) qPCR of wildtype and *Smad4*^{KO} *BRAF*$^{V600E/+}$ tumor organoids show upregulation of integrins (* = p-val < 0.05, Student's *t*-test). B) ChIP-Seq of SMAD4 overlayed with significantly regulated genes in pINDUCER-SMAD4 RNA-Seq from tumor organoids. C) DAVID Analysis of genes upregulated and bound by SMAD4. D) DAVID Analysis of genes downregulated and bound by SMAD4.

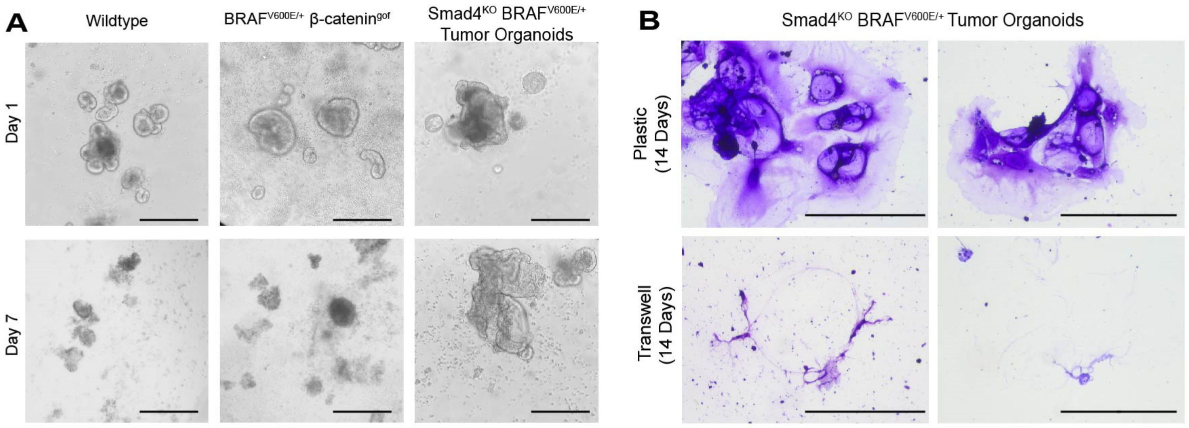

Supplemental Figure S3. Tumor organoids are capable of invasive behavior in 2D enviornment. A) Representative images of organoids grown independent of BME R1 and seeded directly to plastic. Only *Smad4^{KO} BRAF$^{V600E/+}$* tumor organoids show the capability of surviving on plastic after 7 days post-seeding. B) Crystal violet stains of *Smad4^{KO} BRAF$^{V600E/+}$* tumor organoids forming 2D colonies on plastic and post-transwell inflitration. Scale bars = 0.5mm

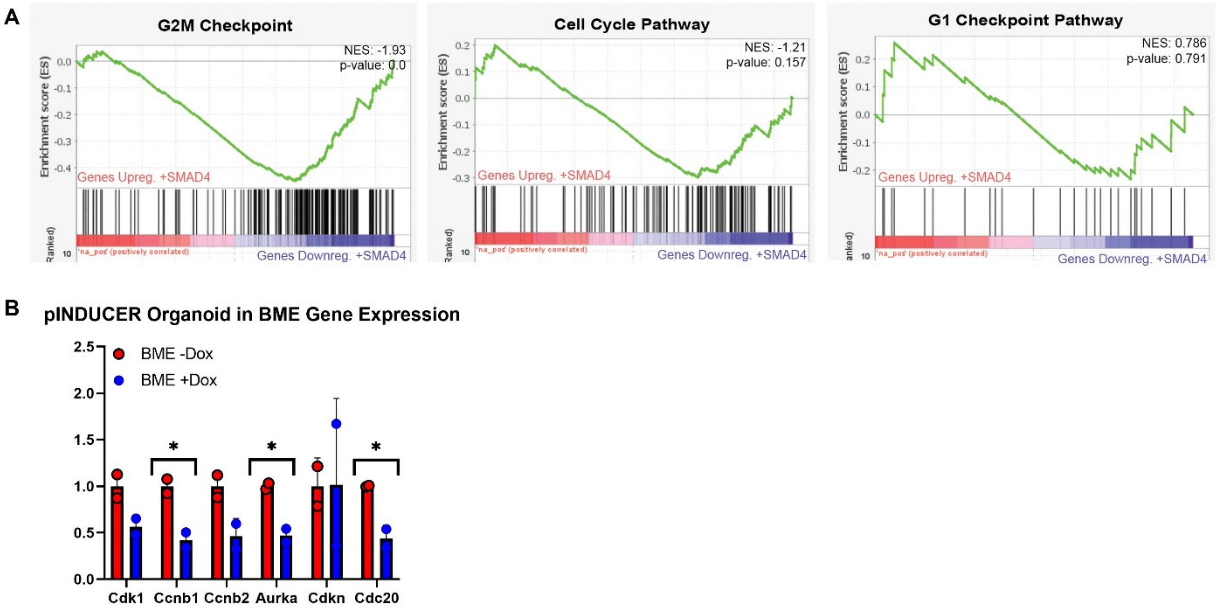

Supplemental Figure S4. Cell Cycle genes are suppressed by SMAD4 in BME R1 culture after delay. A) GSEA analysis of pINDUCER-SMAD4 organoids 48 hours after SMAD4 induction. B) pINDUCER-Smad4 tumor organoids show decrease in cell cycle genes after 5 days post-induction of SMAD4 expression. (* p-value < 0.05, Student's *t*-test).

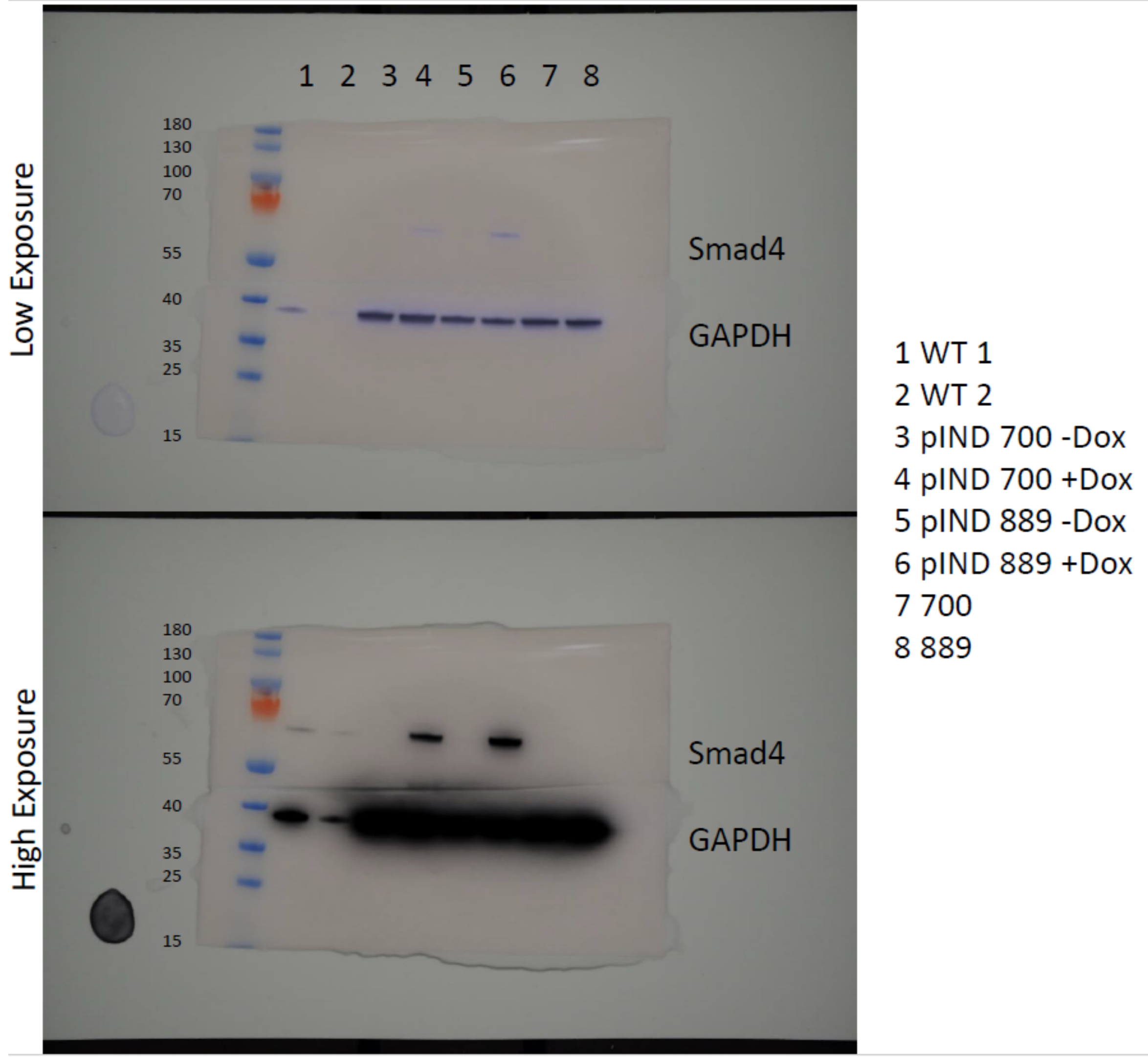

Figure S5: Original blots of Figure 2A
